# Understanding Concentrations of Sexual Violence and Abuse: A New Theory

**DOI:** 10.1177/10778012231189481

**Published:** 2023-07-25

**Authors:** Susan Rayment-McHugh

**Affiliations:** 1 Sexual Violence Research and Prevention Unit, 5333 University of the Sunshine Coast, Brisbane, QLD, Australia

**Keywords:** sexual violence, sexual abuse, concentrations, uneven distribution, theory

## Abstract

Sexual violence is unevenly distributed, with concentrations reported in some contexts and not others, on global, national, and local levels. To advance understanding of why concentrations develop in some settings and not others, existing theory and research is explored and critiqued, identifying a need for new theory generation. Building on existing knowledge, a new theory of contextual activation is proposed, explaining the social and contextual factors associated with the development of sexual violence and abuse concentrations, through the activation at scale of routine causal mechanisms. The paper concludes with a discussion of implications for enhanced prevention and next steps in testing the utility of this new theory.

Sexual violence and abuse (SVA) is a global public health concern impacting millions of individuals worldwide. The devastating impacts of SVA are well documented (Garcia-Moreno et al., 2013; Krug et al., 2002), and there is overwhelming agreement on the need to enhance prevention. The uneven distribution of SVA, however, has received less attention in the literature, despite evidence of higher prevalence rates in some contexts and not others (Rayment-McHugh, 2020). Acknowledging concentrations of SVA reminds us that women and children are at greater risk in some settings (Rayment-McHugh, 2020; Rayment-McHugh et al., 2015a). Better understanding these concentrations and the contexts associated with greater risk may aid prevention and intervention efforts around the world.

## Concentrations of Sexual Violence and Abuse

Epidemiological research reveals extensive variations in the prevalence of SVA on a global level. For example, recent research based on the WHO Global Database on Prevalence of Violence Against Women (Sardinha et al., 2022) found higher lifetime and past-year rates of intimate partner violence (including sexual violence) in low- or middle-income countries and regions. For example, highest lifetime rates were reported in the Oceania (49%) and sub-Saharan Africa (44%) regions, and the lowest rates in central and western Europe (16%/20%) and central Asia (18%). These authors also report variation in prevalence on a country level, with the highest median lifetime prevalence rates reported in Kiribati (53%), Fiji (52%), Papua New Guinea (51%), and Bangladesh and the Solomon Islands (50%), compared to countries with the lowest prevalence rates including Singapore (11%) and Switzerland (11%).

Single-country research also exposes variations in the distribution of SVA at a national level. For example, the highest rates of child sexual abuse (CSA) in India are reported in institutional settings (Bhave & Saxena, 2013); youth gang rape and sexual slavery are reported to be most common in rural areas of the Democratic Republic of Congo (Nelson et al., 2011); female youth in Japan are reported to be at increased risk when using trains to commute, with “train-perverts” exposing themselves or sexually assaulting passengers (Tanaka et al., 2017); in Finland, higher rates of abuse have been documented in Lapland Province, compared to other regions (Sariola & Uutela, 1994); while in Brazil, sexual violence is more prevalent in areas of higher unemployment and economic disadvantage (da Silva & Roncalli, 2018). New Zealand research has revealed higher rates of CSA among Maori women (Fanslow et al., 2007); Vogelman and Eagle (1991) found the likelihood of rape victimization higher for those living in “township areas” in South Africa; whereas in Australia, CSA is most prevalent in remote Aboriginal and Torres Strait Islander communities (Aboriginal Child Sexual Assault Taskforce, 2006; Robertson, 2000; Wild & Anderson, 2007).

Variations in SVA prevalence are also evident at smaller geographic levels. For example, local Australian research identified two communities in Queensland with higher-than-average rates of youth-perpetrated sexual violence. Within these communities, the highest rates were reported in public spaces including in local parks, and behind vacant buildings, particularly at night (Smallbone et al., 2013). An analysis of 800 rape cases, looking for crime patterns in South Africa (Vetten, 1998), provides a further example of micro-level endemic concerns, identifying streets and buildings in Johannesburg where higher numbers of rapes had occurred. Further, higher numbers of rape offenses were linked to some hotels, parks, and train stations. Seminal research by Sherman, Gartin, and Buerger (1989) provides a further example of micro-level variation. They examined over 300,000 police calls for service in Minneapolis over a 12-month period and spatially identified “hot spot” addresses or street intersections, including for rape offenses. More recently, Canadian research examined place characteristics and sexual crime, finding a range of socio-demographic factors associated with higher rates of reported sexual violence including areas with higher numbers of single individuals, areas with more liquor establishments, and areas with more schools (Hewitt et al., 2018).

Within-institutional variations in prevalence of CSA have also been reported. For example, Australian research found disparities in abuse claims between different groups within the Australian Catholic Church. The highest proportion of non-ordained religious members alleged to have perpetrated abuse (40.4%) were within St John of God Brothers, compared to the lowest proportion (0.6 and 0.3%, respectively), within Brisbane's Sisters of St Joseph of the Sacred Heart, and within Sisters of Mercy (Royal Commission into Institutional Responses to Child Sexual Abuse, 2017).

Beyond evidence of the uneven distribution of SVA, analysis suggests that abuse concentrations develop in several different settings. Endemic SVA is regularly reported in international conflict zones (Bastick et al., 2007), as well as in other crisis settings, such as during peacekeeping missions (Nordas & Rustad, 2013), in refugee camps and detention centers (Vu et al., 2014), and following natural disasters (Fisher, 2010). For example, over 350,000 Rwandan (Tutsi) women were raped during the 1994 Rwandan genocide or following this in refugee camps (Bijleveld et al., 2009), while 25,000–50,000 mostly Bosnian Muslim women were raped by Serbian military during the mid-1990s ethnic conflict in Bosnia–Herzegovina (Snyder et al., 2006).

Concentrations of SVA are also reported in isolated or disadvantaged communities such as on Pitcairn Island in the Pacific Ocean. There, nearly half the adult male population was arrested and charged in relation to CSA (Marks, 2008; Power, 2007; Vaughan, 2006). High prevalence of CSA reported in remote Aboriginal communities in Australia serves as another example, with multiple inquiries spanning decades, describing the problem as “massive” and “epidemic” (Aboriginal Child Sexual Assault Taskforce, 2006; Robertson, 2000; Wild & Anderson, 2007). Indeed, official data suggests that Aboriginal women are two to four times more likely to experience SVA (Tarczon & Quadara, 2012), and Aboriginal children three times more likely to be victims of CSA (Aboriginal Child Sexual Assault Taskforce, 2006).

Abuse concentrations have also been reported across institutional settings (Swain, 2014), including religious institutions (Parkinson et al., 2012); schools (Briggs, 2014); sporting clubs (Leahy et al., 2002); university settings (Australian Human Rights Commission, 2017), and in prisons (Wolff et al., 2006). John Jay College's comprehensive study of CSA in the Catholic Church serves as an important example, revealing allegations of abuse from 10,667 victims against 4,392 (4%) priests, between 1950 and 2002 (Terry, 2008), with many others likely to have experienced abuse without making an official report.

## In Pursuit of Understanding

Despite recognition of the uneven distribution of SVA, understanding of how and why concentrations of SVA develop in some contexts and not others remain limited. Advancing this understanding is necessary to inform prevention and responses to this issue. Indeed, there is a growing policy recognition of the need to target and tailor prevention to priority groups most impacted by SVA, but this is dependent on an understanding of the factors that contribute to this phenomenon. Enhanced understanding of why concentrations of SVA occur in some contexts and not others will thus aid development of the most appropriate responses at local, national, and global levels. Wikstrom (2007) sheds light on this issue, describing and critiquing “common pitfalls” in crime prevention practice, where hasty, reactive decisions are made to implement prevention activities, which may not target central causal factors. Instead, Wikstrom (2007) argues for a “knowing-before-doing” or a “knowledge-based” approach to prevention, built on a fuller understanding of relevant causal factors. In this approach, comprehensive understanding of the crime problem, and its local characteristics, directly informs the design and planning of prevention strategies, ultimately enhancing effectiveness. A comprehensive understanding of how and why concentrations of SVA occur is thus necessary for advancing a “knowledge-based” sexual violence prevention agenda, guiding who, what, where, and how to target prevention efforts. Indeed, without this knowledge, policy and practice professionals responsible for enhancing safety in these contexts can, at best, guess at the most appropriate targets (Rayment-McHugh et al., 2015a). To enhance understanding, existing etiological theories of SVA perpetration, existing explanations for endemic SVA concentrations, and an extended knowledge base are explored. An argument is made for new theory to enhance understanding.

### Existing Etiological Theories of SVA Perpetration

Theories have already been developed to explain the perpetration of SVA. For example, the Integrated Life-Course Developmental Theory of Sexual Offending, developed by Smallbone and colleagues (Smallbone & Cale, 2015; Smallbone et al., 2008), proposes that sexual offending is best explained by the interaction between an individual and their immediate environment. They argue that a universal biologically based potential exists among human beings for violent behavior including SVA. Positive developmental and socialization experiences generally constrain this potential and build the motivation and skills for socially responsible behavior and relationships; however, adverse experiences may leave individuals vulnerable to abusive behavior. Importantly, like other integrated theories, Smallbone and colleagues acknowledge the role of contextual factors in facilitating or constraining prosocial or abusive behavior. They argue that this may occur at both systemic (family, peer, neighborhood, and community systems) and situational (opportunity structures and precipitating conditions) levels.

Other etiological theories (Finkelhor, 1984; Marshall & Barbaree, 1990; Ward & Beech, 2006, 2016) are similarly multifactorial, integrating individual, systemic, and situational factors in the explanation of SVA perpetration. Finkelhor's (1984) Four Preconditions Model, for example, identifies four key elements necessary for a sexual offense to occur: a motivated individual; overcoming internal inhibitors (e.g., cognitive distortions); overcoming external inhibitors (e.g., opportunity); and overcoming a potential victim's resistance (Finkelhor, 1984). In turn, Ward & Beech (2006, 2016) offer a “network of causal factors” in their original and revised integrated theory of sexual offending; proposing biological, ecological, neuropsychological, and personal-agency level factors that interact at different levels to contribute to sexual offending behavior.

This body of work explains SVA perpetration by individual people, but largely ignores explicit discussion of endemic concentrations of SVA. Despite this, there are important learnings to be gained from this formative work, which may advance understanding of SVA concentrations. Indeed, a common factor among these theories is an integrated perspective, with multiple contributing factors, acknowledging both heterogeneity and complexity in SVA perpetration. Notably, these integrated theories highlight both individual and contextual factors in explanation and in particular, the interaction between an individual and their environment. For example, Marshall & Barbaree (1990) state that “certain environmental factors interact with particular states of the individual to facilitate sexual aggression and abuse” (p. 268).

At an individual level, personal dysregulation has been linked with sexual offending behavior (Ward & Beech, 2006) through a developmental lens (Smallbone et al., 2008), widely supported by empirical research (Ha & Beauregard, 2016). This includes emotional and behavioral regulation problems, intimacy deficits, offense-supportive cognitions, and sexual interest (Ward & Beech, 2016). In turn, contextual (systemic and situational) factors facilitate or constrain potential for abusive behavior by an individual. At a systemic level, the influence of cultural norms and values, conveyed through family, peers, workplaces, or community settings, are acknowledged (Smallbone et al., 2008), while links between neighborhood poverty, unemployment, and housing stress have been linked empirically with child maltreatment (Freisthler et al., 2006). The capacity of families, bystanders, and institutions to provide capable guardianship also impacts levels of protection (Smallbone et al., 2008). On a situational level, integrated theories have highlighted the role of opportunity (Finkelhor, 1984) and routine activities (Smallbone et al., 2008) on abuse perpetration. The potential for guardians and place managers to provide protection is also acknowledged (Smallbone et al., 2008), with empirical support (Leclerc et al., 2015; Cook et al., 2021).

### Existing Explanations for Endemic SVA

No previous published theories have explicitly attempted to explain concentrations of SVA across diverse contexts. This is a notable limitation, given context-specific explanations may not advance understanding of SVA in other diverse settings. Indeed, existing theories of SVA concentrations are context-specific, with most attention given to explaining rape in war or conflict settings. The most influential of these is the Strategic Rape Theory, a feminist theoretical conceptualization based on notions of gendered and politicized power (Kirby, 2013). Put simply, Strategic Rape Theory contends that military leaders use rape as a weapon of war to achieve strategic military goals, just as they would use guns or bombs (Card, 1996; Farwell, 2004; Skjelsbaek, 2001). Importantly, Strategic Rape Theory has had wide reaching impacts, including contributions to the inclusion of sexual violence as a war crime, a crime against humanity and as an act of genocide (Davies & True, 2015). It is also recognized within the 1998 Rome Statute of the International Criminal Court, and supported by other United Nations (UN) resolutions, for example, the UN Security Council Resolution 1820 (adopted in 2008), which calls for an end to the intentional use of sexual violence as a war tactic and to impunity for SVA perpetrators (United Nations Security Council, 2008, p. 1 and 3). Despite its wide acceptance and influence, however, Strategic Rape Theory has been criticized for a lack of evidence supporting deliberate intent (Cohen et al., 2013; Maedl, 2011) and shortcomings in explaining variations in SVA prevalence across different wars and conflicts (Cohen, 2013; Cohen & Nordas, 2014; Wood, 2014). For example, a research into African conflicts between 1989 and 2009 found that 64% of armed groups did not engage in SVA (Cohen et al., 2013). Wood (2006) also reports the extent of sexual violence varying over the period of a conflict, while Alexandre & Mutondo (2022) suggest wartime SVA reflects social attitudes toward women outside of war contexts. For these reasons, Strategic Rape Theory falls short of explaining the development of endemic SVA concentrations in some contexts and not others.

The Strategic Rape Theory has its foundations in Feminist Theory, which emphasizes gender inequality, and gendered power imbalance, and the societal structures that create or reinforce this, as causal factors for male perpetrated SVA (Brownmiller, 1975). Applied to conflict settings, Feminist Theory proposes that war increases opportunities for men to dominate women. Feminist Theory does not, however, specifically distinguish between endemic and nonendemic SVA contexts, nor does it explain variations in SVA prevalence across different conflict settings. Thus, while a gendered analysis aids our understanding of this issue, this alone does not sufficiently explain the development of endemic SVA concentrations in some contexts and not others.

Integrated theories of SVA in conflict settings have also been proposed, though with seemingly less policy or practice impact than Strategic Rape Theory. Henry et al.'s (2004) Multifactorial Modal of Wartime Rape, for example, builds on these previous conceptualizations, advocating the collective interplay of socio-cultural, individual (e.g., childhood experiences) and situational (e.g., war context) factors provides a more comprehensive account of sexual aggression in conflict zones. Similarly, Gottschall (2004) proposed a Biosocial Theory of rape in war zones, integrating biological and social-cultural factors to explain this phenomenon.

No published theory currently accounts for the diversity in endemic settings, or advances understanding of generic causal factors linked to abuse concentrations. Despite the context-specific focus of these existing theories, however, there are key learnings that may aid a broader understanding of SVA concentrations. Indeed, what these theories have in common is the fundamental role of contextual factors in the explanation of endemic SVA concentrations.

While research literature on SVA concentrations has largely focused on problem identification and description, and international responses and advocacy, rather than explanation (Cohen et al., 2013), Rayment-McHugh et al. (2015b) identified common contextual factors in an analysis of five discrete case studies involving endemic SVA concentrations (international conflict zones; Abu Ghraib Prison [Iraq]; remote Aboriginal Communities in Australia; Pitcairn Island; and the Catholic Church), that appear central to explaining this phenomenon.

On Pitcairn Island, for example, early concerns raised about CSA were ignored, reflecting a breakdown in controls that should have prevented the development and persistence of this behavior. A culture of male entitlement was also evident, with girls not believing they could prevent abuse (Marks, 2008; Power, 2007). At Abu Ghraib Prison, accused soldiers experienced numerous environmental pressures and stressors that may have contributed to their out-of-character behavior, including overwhelming workload and safety fears and limited training and supervision (Zimbardo, 2007). They had also been advised that different rules applied to their prisoners and to “set the physical and mental conditions for favorable interrogation of witnesses” (Taguba, 2004, p. 18). Collectively, such conditions may have provoked, pressured, and suggested permissibility for abusive behavior. As a further example, reports of abuse in the Catholic Church exposed a culture of denial and a failure of the organization to respond appropriately to abuse allegations, suggesting a breakdown in organizational safety controls that enabled the persistence of abuse.

Importantly, and common across all five case studies, the coincidental presence of large numbers of individuals highly motivated to offend could not adequately explain endemic SVA concentrations. This was most apparent at Abu Ghraib Prison, where soldiers who perpetrated abuses had been subject to psychological screening to eliminate preexisting predispositions or psychopathology (Zimbardo, 2007). Similarly, Terry's (2008) study of CSA within the Catholic Church in the USA uncovered a late-onset of abusive behavior, with most priests offending approximately 11 years following ordination, contradicting the likelihood of deviant intent when joining the clergy. Rayment-McHugh et al. (2015b) point out that thousands of pedophiles did not happen to join the Catholic Church in the USA in the 1960s–1970s, nor did thousands of sexually deviant individuals involve themselves in the Rwandan conflict in the 1990s. Instead, and consistent with the etiological theories outlined above, context seems fundamental to SVA concentrations, necessitating a central role in any explanatory theory.

### Extending the Knowledge Base

There are important gaps in the knowledge base currently informing explanations for endemic SVA concentrations. Indeed, most existing knowledge of abuse concentrations developed independently of the nonendemic SVA field (evidenced by few citations to such work). In turn, the main body of literature informing understanding, prevention, and responses to (nonendemic) SVA ignores explicit discussion of endemic concentrations (Rayment-McHugh et al., 2015b). Extending this knowledge base, by applying previously under-utilized criminological and social psychological perspectives may provide further insight into SVA concentrations.

Crime concentrations and “hot-spots” have received considerable attention in the general criminological literature, so applying a criminological lens may help to advance understanding. This literature has largely (until fairly recently) overlooked SVA; however, it provides important insight. Criminological theories developed to explain (non-SVA) crime concentrations include Social Disorganization Theory (Shaw & McKay, 1969) and Collective Efficacy Theory (Sampson et al., 1997). These two criminological theories highlight the importance of informal social controls and “collective efficacy” (capacity of neighborhoods to establish informal social controls to achieve common community goals) as mediators of crime, with structural factors like poverty, family dissolution, and ethnic heterogeneity hypothesized to weaken these controls.

The discipline of environmental criminology is also concerned with the influence of environmental factors on crime commission. Routine Activities Theory (Cohen & Felson, 1979) proposes that people respond to crime opportunities within their usual daily routines, and that crime occurs when a motivated person and a vulnerable victim come into contact in the absence of a capable guardian. Routine opportunity is central to this theory and necessary for crime to occur. Extending this work, Wortley (1997, 1998, 2001) suggests that situations are more than simply passive opportunities in which an offense may occur, proposing instead that features of the immediate situation may precipitate offending motivations, in addition to the more dominant view, that an already motivated person seeks out opportunities for crime/abuse. Guardians, including family members, peers, or bystanders, in turn exert informal social control that may regulate offending behavior (Eck, 2003). For example, the mere presence of a potential guardian may increase the risk of detection, and thus deter crime, while active guardianship may interrupt abusive behavior or protect “potential victims.” Collectively, environmental criminology theories are based on three key premises: (a) environmental factors shape criminal behavior; (b) offenses concentrate under conditions that facilitate this behavior; and c) altering criminogenic environments can alter behavior (Wortley & Mazerolle, 2008).

Social psychology research further supports a person-situation paradigm, and provides additional insight into contextual influences on human behavior. For example, foundational social psychological research of the 1960s and 1970s showed people's willingness to inflict harm on others under pressure from an authority figure (Milgram, 1974); compromised self-restraint when group conditions override individual identity (deindividuation) (Zimbardo, 2007); and the conditions under which dehumanization may disinhibit aggression (Bandura et al., 1975). This shows the capacity of otherwise “normal” people to act in “abnormal” ways; described by Arendt (1963) as the “banality of evil.” More recently, Zimbardo (2007) also challenged assertions that necessarily label those involved with harmful behavior in poor light. Instead, in what has become known as the “bad apple vs bad barrel” debate, Zimbardo highlights the negative impacts of criminogenic-environmental conditions on individual behavior, and thus the role of the “bad barrel” in explaining some behavior.

### Need for a New Theory

What is needed, therefore, is a more cohesive theory of endemic SVA concentrations that explains causal factors and the conditions under which these concentrations develop, and an ability to account for variations that occur across different settings. Informed by the above review, a contextual activation theory is proposed, explaining the social and contextual factors associated with the development of SVA concentrations, including why they might develop in some contexts and not others. Its broad focus encompasses the diverse settings in which this occurs, and the complexities of this phenomenon.

## A Contextual Activation Theory of Endemic SVA

### Guiding Framework and Theory Overview

Informed by thoughts on causal explanation advanced by realist scientific philosophy (Bhaskar, 1978; Harre, 1972), a realist social explanatory framework (Pawson & Tilley, 1997) guides development of this proposed theory. Based on a generative model of causation, the realist approach posits that “causal outcomes follow from mechanisms in context” (Pawson & Tilley, 1997, p.58). “Mechanisms” are the processes that produce certain behavior (outcomes), while “context” refers to the environmental conditions that allow the activation of causal mechanisms. In the case of complex social issues, such as SVA, context and mechanisms can sometimes be difficult to distinguish, with meaning best achieved through the construct's explanatory role (Pawson, 2013). Outcomes (in this case endemic SVA) occur as a result of the actions of specific mechanisms activated within specific contexts (Pawson & Tilley, 1997). It is argued that meaningful explanation and understanding depends on “identifying causal mechanisms and how they work, and discovering if they have been activated and under what conditions” (Sayer, 2000, p. 14).

The World Health Organization defines sexual violence as “any sexual act, attempt to obtain a sexual act, unwanted sexual comments or advances, or acts to traffic, or otherwise directed, against a person's sexuality using coercion, by any person regardless of their relationship to the victim, in any setting” (Krug et al., 2002, p. 149). Fundamentally, this same behavior constitutes SVA, irrespective of location or prevalence. The same primary causal mechanisms, therefore, are likely to contribute to this behavior. Instead, the key factor accounting for difference in prevalence rates is the quantum of affected people at a given place and time. It is thus proposed that social and contextual factors may influence the behavior of large numbers of people in certain settings, contributing to concentrations of SVA. It is theorized, therefore, that under conducive environmental conditions (context), the factors that contribute to nonendemic SVA (mechanisms) may be activated at scale, leading to higher-than-usual prevalence rates (concentrations of SVA). Proposed mechanisms and contextual conditions are discussed separately and summarized in Table 1.

**Table 1.** Key Mechanisms and Contextual Conditions.

Mechanisms	Situational precipitants	Situational cues that elicit offending motivations and weaken personal controls over behavior, including prompts, pressures, permissibility, and provocations
	Breakdown in personal or informal social regulatory controls	Transitory or chronic breakdown in personal controls over behavior, e.g., behavioral and emotional dysregulation or cognitive distortions excusing SVA
		Transitory or chronic breakdown in available guardianship and supervision that would otherwise protect or prevent an offense
	Opportunities	Situations in which potential offenders come into contact with potential victims without a capable guardian
Contextual conditions	Social conditions that support SVA	Norms, values, and beliefs that support SVA, e.g., cultural norms and values regarding gender and interpersonal relationships, or widespread exposure to violence that normalizes this behavior
	Breakdown in formal regulatory controls	Transitory or chronic breakdown in formal controls that establish, enforce, and monitor appropriate standards of behavior, including compromised law, law enforcement, and protection services, resources, and policies.
	Population stressors	Population stressors

### Mechanisms

Mechanisms are “underlying entities, processes, or structures which operate in particular contexts to generate outcomes of interest” (Astbury & Leeuw, 2010, p. 368). With respect to complex social issues, multiple concurrent mechanisms typically operate in parallel (Bunge, 2004). Building on existing etiological theories with a contemporary application of criminological perspectives provides a new way to conceptualize individual and situational factors that contribute to abuse. Stemming from this, it is proposed that SVA may be triggered by precipitating cues eliciting offending motivations within a given situation; a breakdown in personal (self-regulation) or informal social (guardianship, supervision) regulatory controls subsequently means these motivations are not constrained, while mostly routine convergence settings create opportunities for “potential offenders” to come into contact with “potential victims” in the absence of capable guardians. This theory, therefore, proposes three primary causal mechanisms for SVA: (a) situational precipitants; (b) breakdowns in usual controls over behavior; and (c) opportunity for SVA. Significant diversity within these overarching causal mechanisms is acknowledged.

#### Situational Precipitants

Wortley (1997, 1998) describes situational precipitants as the factors within the immediate environment (situation) that elicit offending motivations. Four different precipitants are proposed: prompts, pressure, permissibility, and provocation.

Prompts are situational cues that tempt or stimulate someone to offend, and comprise situations that model abusive behavior, such as exposure to the sexual behavior of others. The influence of such behavioral models is well documented. For example, social learning theory explains how human behavior is influenced by the actions of others and through observing the response to this behavior (Bandura, 1977). Pervasive exposure to SVA, for example, is likely to have a similar effect.

Social pressure refers to the real or perceived pressure experienced by an individual to behave in a certain way, in a given situation. Social psychology research provides many examples of this, including Asch's (1955) famous experiments on conformity to group norms, where the participants provided incorrect answers to a basic question about the length of a line on a card, influenced by the (incorrect) response given by a researcher posing as another participant.

Permissibility refers to the weakening of usual personal controls and moral restraint, in response to cues within a situation that are perceived as “permitting” the behavior. Deindividuation and dehumanization are two psychological processes that might contribute to such permissibility (Bandura et al., 1975; Zimbardo, 2007). Instances of dehumanization are evident in international atrocities. For example, during the Rwandan genocide, Tutsi men and women were disrespectfully called “cockroaches,” while during the Holocaust, Jewish people were described in equally offensive terms (Waller, 2007). Perceived encouragement from co-offenders is another example.

Finally, provocations involve the features of a situation that trigger stress or adverse emotional arousal, which may in turn provoke an offense. Examples include sexual or nonsexual frustration.

#### Breakdown in Personal or Informal Social Regulatory Controls

Skills used by individuals to regulate their behavior (personal regulatory controls) include emotional and behavioral regulation, empathy, social skills, cognitive reasoning, and perspective taking (Smallbone & Cale, 2015). Failure to engage these skills may lead to impulsive, poor, or even harmful behavior. Etiological theories of routine (nonendemic) SVA perpetration have associated personal dysregulation with sexual offending behavior (Smallbone et al., 2008; Ward & Beech, 2006; Ward & Hudson, 2000), with these links supported by empirical research (Ha & Beauregard, 2016). Alcohol or drug use may also disinhibit behavior (Kraanen & Emmelkamp, 2011).

Behavior may also be regulated through informal social control provided by potential guardians, such as family members, peers, bystanders, or those with supervisory responsibilities in designated settings (Eck, 2003). Effective guardianship requires availability, a willingness to monitor, context-specific knowledge to help assess observed behavior, and willingness to take some form of action (Reynald, 2010). Potential guardians may actively protect potential victims, interrupt abusive behavior, or increase the likelihood of detection. A breakdown in informal social control occurs when guardianship is unavailable or ineffective (regardless of the reason). Research reveals the factors that may impact potential guardians’ willingness to intervene including perceived competence to take action (Huston et al., 1981), the primacy of responsibility (Felson, 1995), and diffusion of responsibility in the presence of other potential guardians (Darley & Latane, 1968).

#### Opportunity

Finally, crime cannot occur without opportunity (Felson & Clarke, 1998), making this an essential component of any theory. Clarke (2012) contends that “the more opportunities for crime that exist, the more crime there will be” (p. 6). Opportunities for SVA may emerge within a given situation or setting or may be manipulated by a highly motivated individual. The most criminogenic situations allow “potential offenders” access to “potential victims,” with limited risk of detection or punishment. Thus, it is proposed that settings with SVA concentrations provide greater opportunities for SVA to occur—influencing motivations to offend, increasing vulnerabilities among “potential victims,” and impairing potential guardianship.

### Context

This contextual activation theory contends that endemic SVA may be contingent on contextual factors that trigger causal mechanisms to a greater-than-usual degree within given populations at given times. Accordingly, it is proposed that nonendemic and endemic SVA are distinguished by the environmental conditions conducive to increased rates of abuse. The environmental conditions that trigger causal mechanisms for crime may be considered criminogenic. What makes a setting criminogenic is thus the extent to which it triggers causal mechanisms for crime. The criminogenic-environmental conditions proposed to increase the prevalence of SVA include: (a) social conditions supporting SVA; (b) a breakdown in formal regulatory controls; and (c) population-level stressors.

#### Social Conditions that Support SVA

Social context communicates norms, values, and behavioral expectations (Bronfenbrenner, 1979), which in turn influences behavior, including sexual behavior, particularly where these messages relate to gender and interpersonal relationships. Early research into “rape-prone” communities revealed gender separation, power imbalance, and a tolerance for violence, as the key social conditions contributing to high prevalence of rape (Sanday, 1981). Where people are regularly exposed to violence and sexual violence, they may become desensitized to such behavior, resulting in the normalization of SVA. Rigid gender roles, gender norms that promote male power and entitlement, and/or norms that justify violence toward women, have all been linked to abuse (Heise et al., 1999, Krug et al., 2002).

#### Breakdown in Formal Regulatory Controls

Laws and legislation, police practice, justice system processes, and statutory child protection services are all examples of formal external regulatory systems designed to establish, monitor, and enforce appropriate standards of behavior. These systems are intended to protect individuals from abuse, communicate behavioral expectations, and set clear consequences for SVA.

Where these systems are absent or poorly developed, compromised or dysfunctional in some way, a reduction in the rules and laws guiding behavior, fewer consequences for SVA, and less external scrutiny may occur. The absence of laws or practices explicitly penalizing SVA, and/or a failure of formal systems to respond effectively, can tacitly imply that such behavior is acceptable. Weak or absent laws and government policies related to SVA, along with ineffective policing and judicial systems, therefore, increase the likelihood of SVA occurring (Krug et al., 2002).

#### Population Stressors

In contrast to personal stressors (e.g., loss of a family member, divorce, unemployment) that affect discrete individuals, population-level stressors impact whole populations. Stress or fear, for example, may be experienced by both soldiers and civilians in active conflict zones (Ford, 1999); stress linked to poverty may be experienced in some marginalized communities (Krug et al., 2002), while intergenerational trauma may be experienced by colonized and dispossessed populations (Robertson, 2000). Such stressors may in turn trigger negative mood states and out-of-character behavior.

Widespread poverty and social disadvantage are also linked empirically to high crime rates (Pratt & Cullen, 2005). For example, economic pressures may create the conditions that increase opportunity for SVA, such as having to work in isolated fields, or that may impair the availability of informal social controls, with potential guardians engaged in work or other activities to obtain food and shelter (Krug et al., 2002).

### Summary

Building on the theory and research outlined earlier, this theory of contextual activation explains how social and contextual factors may contribute to the development of SVA concentrations in some contexts and not others, specifically where social conditions support abuse, formal regulatory controls are compromised or dysfunctional, and stressors impact whole populations. It is theorized that in these contexts, a greater-than-usual proportion of the population may be motivated to engage in SVA behaviors, with an associated breakdown in “possible” controls over conduct in the face of opportunity, facilitating SVA at scale. It is thus an attempt to explain “how” social and contextual factors may contribute to SVA concentrations, rather than simply providing a so-called ingredient list of relevant social and contextual influences. Accordingly, it is proposed that “drivers” contributing to SVA in other routine, nonendemic contexts may be activated at scale, resulting in SVA concentrations. In essence it is a “bad barrel” theory, with potentially criminogenic contextual conditions providing an important explanatory function.

## Implications for Prevention

Importantly, this theory offers new opportunities for prevention. Having a thorough understanding of the “problem” is fundamental to the “knowing-before-doing” approach to prevention advocated by Wikstrom (2007). The theory contributes to prevention, therefore, by identifying both key causal mechanisms and the contextual conditions that contribute to SVA concentrations, to inform prevention policy and practice.

Two key areas of guidance are noteworthy. Firstly, given that most existing efforts to prevent endemic SVA concentrations are focused on addressing conducive contextual conditions, additional targets for prevention are presented, by identifying the role of key causal mechanisms in this problem. The call to “end impunity” for SVA perpetrators, for example, has become somewhat synonymous with prevention of sexual violence in international conflict zones. Justice system reforms needed to facilitate this are indeed important; however, this new theory suggests that there is more that can be done beyond strengthening formal regulatory controls. Indeed, on its own, this is unlikely to be sufficient to tackle such a pervasive and complex problem. Efforts to address gender inequality are also currently advocated (WHO, 2009), and form another crucial component of a comprehensive response to this problem. In accord with the theory, however, prevention efforts could be further extended by addressing a range of population-level stressors, as well as attending to identified causal mechanisms. Building prevention initiatives to address causal mechanisms would involve a targeted focus on immediate situations to reduce precipitating conditions and opportunity, as well as strengthening informal social controls through enhanced guardianship, and promotion of self-regulation skills. It is possible that this may have a more immediate impact than focusing only on distal contextual influences, given the length of time they may require to impact change. For example, legislative drafting and other justice system reforms can take years to implement, whereas strengthening informal guardianship in identified “hot-spots” may produce rapid results.

Secondly, the theory's submission that casual mechanisms may be indeed shared across both endemic and nonendemic settings suggests that effective prevention efforts in more routine, nonendemic settings may also be relevant and effective for preventing endemic SVA concentrations. In doing so, the theory reveals new opportunities for prevention, based on existing evidence from the nonendemic SVA field. Situational crime prevention (targeting immediate situations that impact behavior) and developmental prevention (addressing early personal risk factors), for example, have direct relevance here. Each has a strong evidence base proving effectiveness in crime prevention (Smallbone & McKillop, 2015).

Directly informed by this theory, a comprehensive approach to the prevention of endemic SVA concentrations may now be adopted. The organizing framework proposed by Smallbone & Rayment-McHugh (2017) is used to integrate the range of potential prevention strategies in a user-friendly manner. This nine-point prevention matrix combines primary, secondary, and tertiary levels of prevention based on the public health model, with specific prevention targets identified within the so-called “crime-triangle” (Eck, 2003), namely, “offenders” (or “potential offenders”), “victims” (or “potential victims”), and offense settings. Combined, this produces nine areas for attention in prevention-planning and implementation, ensuring overall that a comprehensive response is provided. Example prevention strategies informed directly by this new theory and spanning both contextual conditions and causal mechanisms are outlined in Table 2. Importantly, Smallbone & Rayment-McHugh (2017) suggest that this matrix be used to map existing approaches and identify gaps in prevention practice to guide prevention-planning at global, national, or local levels, or even within discrete organizations, as well as to guide research prioritization.

**Table 2.** Prevention Options Informed by the New Theory.

	Primary prevention	Secondary prevention	Tertiary prevention
(Potential) offenders	Law & policy reforms; general deterrence; developmental prevention; sexual ethics programs	Reinforce behavioral standards; developmental prevention; helplines	Offender rehabilitation & risk management; specific deterrence; selective incapacitation
(Potential) victims	Law & policy reforms; raise awareness; responsible bystander training; education programs; enhanced guardianship	Address barriers to disclosure; risk awareness training; targeted guardianship	Preventing re-victimization; “cocooning” high-risk victims; intensive guardianship at specific hot-spots
Settings	Establish healthy social norms; address gender inequality; address disadvantage; housing, education, employment programs; create safe places for women & children; community capacity building; parent education; designing safe organizations	Reduce exposure to violence; challenge norms conducive to SVA; safer recruitment strategies; situational crime prevention (SCP) in at-risk places; safety planning in at-risk places	Organizational/military leadership; programs to address core stressors; SCP at specific hot-spots; increasing surveillance at hot-spots

## Next Steps

Lewin (1945) declared that “nothing is as practical as a good theory” (p. 129). The point being made is that theory advances knowledge and insight, and informs practice. The theory proposed in this paper takes an important step in this direction, although at present, it is only a conceptual proposition for explaining the role of social and contextual factors on the uneven distribution of SVA, and the development of sexual abuse concentrations in some settings and not others. It draws heavily on evidence spanning psychology and criminology disciplines, extending existing feminist explanations, and is informed directly by existing empirical research and theory on both endemic and nonendemic SVA. This new theory, however, now requires testing in real world settings. Indeed, according to Bhaskar (1978), “theory without experiment is empty” (p. 191). Research is now needed to test the relevance of these ideas in the diverse range of settings in which endemic SVA is reported, as well as to test the theory's ability to predict or distinguish endemic and nonendemic settings. As a first step, this could include an examination of the application of this new theory to disparate endemic case studies. Extending that further, a comparison of endemic and nonendemic SVA settings should be considered. Finally, it is hoped that this theory will also serve as a reminder and call to arms for “mainstream” SVA professionals and researchers to think more about abuse concentrations and the role they may play in addressing this complex problem.
